# Supplementary material for: Advancing Lithium-Ion Batteries’ Electrochemical Performance: Ultrathin Alumina Coating on Li(Ni0.8Co0.1Mn0.1)O2 Cathode Materials
Source: Micromachines (Basel). 2024 Jul 9;15(7):894. doi: 10.3390/mi15070894 (PMC11278549; doi:10.3390/mi15070894)
Supplement: Supplementary file 1 [file micromachines-15-00894-s001.zip › micromachines-3059812-supplementary.pdf]

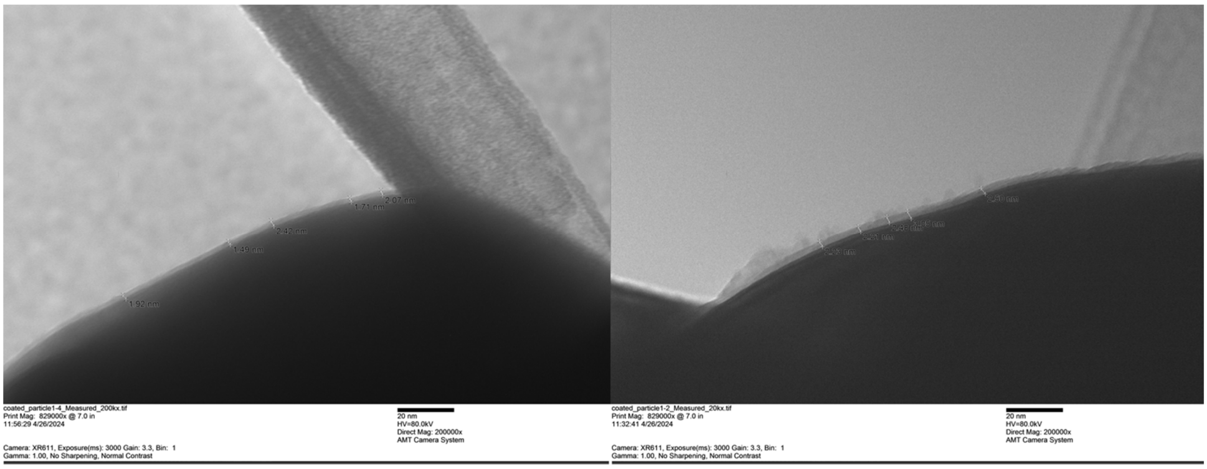

Figure S1. Al_2O_3 ALD Coating thickness from two different locations of NCM811@Al_2O_3 particles.
